# How has medical student learning changed with the pivot to online delivery of ophthalmology in the pandemic?

**DOI:** 10.1371/journal.pone.0282829

**Published:** 2023-03-30

**Authors:** Joan Ní Gabhann-Dromgoole, Conor C. Murphy, Fiona Boland, Andrea J. Doyle, Teresa Pawlikowska

**Affiliations:** 1 School of Pharmacy and Biomolecular Sciences, RCSI, University of Medicine and Health Sciences, Dublin, Ireland; 2 Dept of Ophthalmology, Royal Victoria Eye and Ear Hospital (RVEEH), RCSI, University of Medicine and Health Sciences, Dublin, Ireland; 3 Data Science Centre, RCSI, University of Medicine and Health Sciences, New York City, New York, United States of America; 4 SIM Centre for Simulation Education and Research, RCSI, University of Medicine and Health Sciences, New York City, New York, United States of America; 5 Health Professions Education Centre (HPEC), RCSI, University of Medicine and Health Sciences, New York City, New York, United States of America; AIIMS: All India Institute of Medical Sciences, INDIA

## Abstract

**Purpose:**

This study aimed to measure stakeholder satisfaction with our usual delivery format, which previously relied on a blend of didactic lectures and clinical skills sessions compared to a revised format, which had more emphasis on online learning. We hypothesised that the online flipped classroom (OFC) would facilitate delivery of content in the wake of the pandemic, and result in improved levels of student satisfaction and knowledge gain.

**Design:**

Non randomised intervention study. Group 1 = Traditional delivery (TD) and Group 2 = OFC group.

**Methods:**

A validated course evaluation questionnaire (CEQ) compared perspectives of teaching faculty (n = 5) and students with the traditional delivery (TD) of the 4^th^ year ophthalmology clinical attachment and an OFC approach (TD n = 129 v OFC n = 114).

**Results:**

The OFC group (n = 114; response rate = 24.6%) reported significantly reduced satisfaction with staff motivation of students and provision of feedback, compared to TD (n = 129; response rate = 17.8%). OFC students also felt it was harder to determine what standard of work was expected and found the course less beneficial at helping develop problem-solving skills. Students were dissatisfied with the level of choice afforded by the OFC, specifically how they would learn and assessment options. No significant difference in exam score was observed between the TD and OFC groups. For faculty (n = 5), there was no evidence of a difference between OFC and TD.

**Conclusions:**

Students indicated a preference for the TD compared to the OFC approach. However, both delivery approaches led to comparable student performances as determined by MCQ examination.

## Introduction

The global lockdown and government mandated social distancing measures enforced following the COVID-19 pandemic required that the clinical attachment Ophthalmology module be delivered online. This pivot to the online environment during the COVID-19 pandemic incorporated the flipped classrooms (FC) approach and offered a student-centred learning environment where the traditional face-to-face teaching pedagogy was transitioned into an online distance module using the Blackboard Collaborate (BBc) platform [1]. A pre pandemic review into the effectiveness of a FC concluded that the approach is a promising teaching strategy that can be utilised to promote learner engagement and motivation [6], however, there is little evidence to correlate the implementation of the FC and learner outcomes. In this study, the FC approach was adopted not only as a means to deliver content but also because it afforded the opportunity to engage students in this online environment, enhance problem solving skills and to promote deep learning [2]. Overall, in line with Blooms taxonomy and the FC pedagogy our revised module aimed to move from lower order to higher order cognition [3].

Introduction of technology is not an entirely new concept in medical education or ophthalmology, where studies have previously examined the effectiveness of video webcasts [4], and how digital learning compared to traditional didactic teaching interventions [5]. While there has been increasing interest in the use of a FC approach in medical education [6] and ophthalmology [7–11] there is controversy in the literature regarding this form of teaching particularly with regard to demonstrating positive changes in knowledge and skills. Several systematic reviews have investigated the efficacy of flipped classrooms [6, 12, 13], and while many studies reported the effectiveness of the flipped classroom in terms of learner outcomes categorised according to Kirkpatrick’s framework [14] and Blooms taxonomy, the varying methods for implementation have produced varying results [13]. Additionally, there is a paucity of research detailing changes in these aspects with respect to the use of FC to provide a means of providing continued education for ophthalmology students during the pandemic.

A review of developments in medical education in response to the COVID-19 pandemic [15, 16] found that seven studies described utilising the FC educational intervention [17–23] BEME Guide No 63 identified two studies describing the use of a combination of videoconferencing, FC (with question-and-answer time), video review of surgical procedures, and surgical simulators [21, 22] and BEME Guide No 64 identified further studies which described the incorporation of active learning strategies in response to the pandemic including FC [17, 19, 23]. One study by Sud *et al* described their pivot of undergraduate ophthalmology [20] and positive student feedback from this intervention in terms of ease of accessing material, asynchronous nature and ability to view multiple times. The student feedback also detailed some negative aspects of the intervention related to the diminished interactivity of this approach and the increased time for any doubts to be clarified. However, they do not detail any changes in knowledge gain or skill acquisitions.

There is significant interest in, and positives attributed to the FC, however, Diel *et al* suggest that further work is required to assess the efficacy of the FC approach for ophthalmology in a completely virtual setting, as necessitated by COVID-19 physical distancing constraints [24].

In response to the consequences of the COVID-19 pandemic, we pivoted a didactic and clinical ophthalmology module to an online flipped classroom approach encompassing the same learning objectives for 4th year medical students. We hypothesised that the flipped classroom delivery would result in more engagement with the material, better problem solving and so impact positively on their learning. Thus, as the literature regarding an entirely online flipped classroom approach for ophthalmology is limited our study is timely as it examines the perceptions of both students and faculty with the online FC pedagogy, representing Kirkpatrick–level 1- reaction [25]. Additionally, the evaluation of the impact of introducing this pedagogical model had on knowledge gain was determined, representing Kirkpatrick Level 2- learning [26].

## Methods

### Ethics

This study was reviewed and approved by the Research and Ethics Committee (REC) of the RCSI, University of Medicine and Health Sciences and was conducted according to the principles expressed in the Declaration of Helsinki. Written informed consent was be obtained from all participants (REC 202006015).

### Module description

Our objective was to consistently address the learning outcomes (S1 Appendix) despite the change in delivery supporting constructive alignment whilst responding to the changes precipitated the by COVID-19 pandemic regarding social distancing etc (REF Irish Government Guidelines on closing in March 2020).

### Subjects and study design

#### Study populations

**Fig 1:**
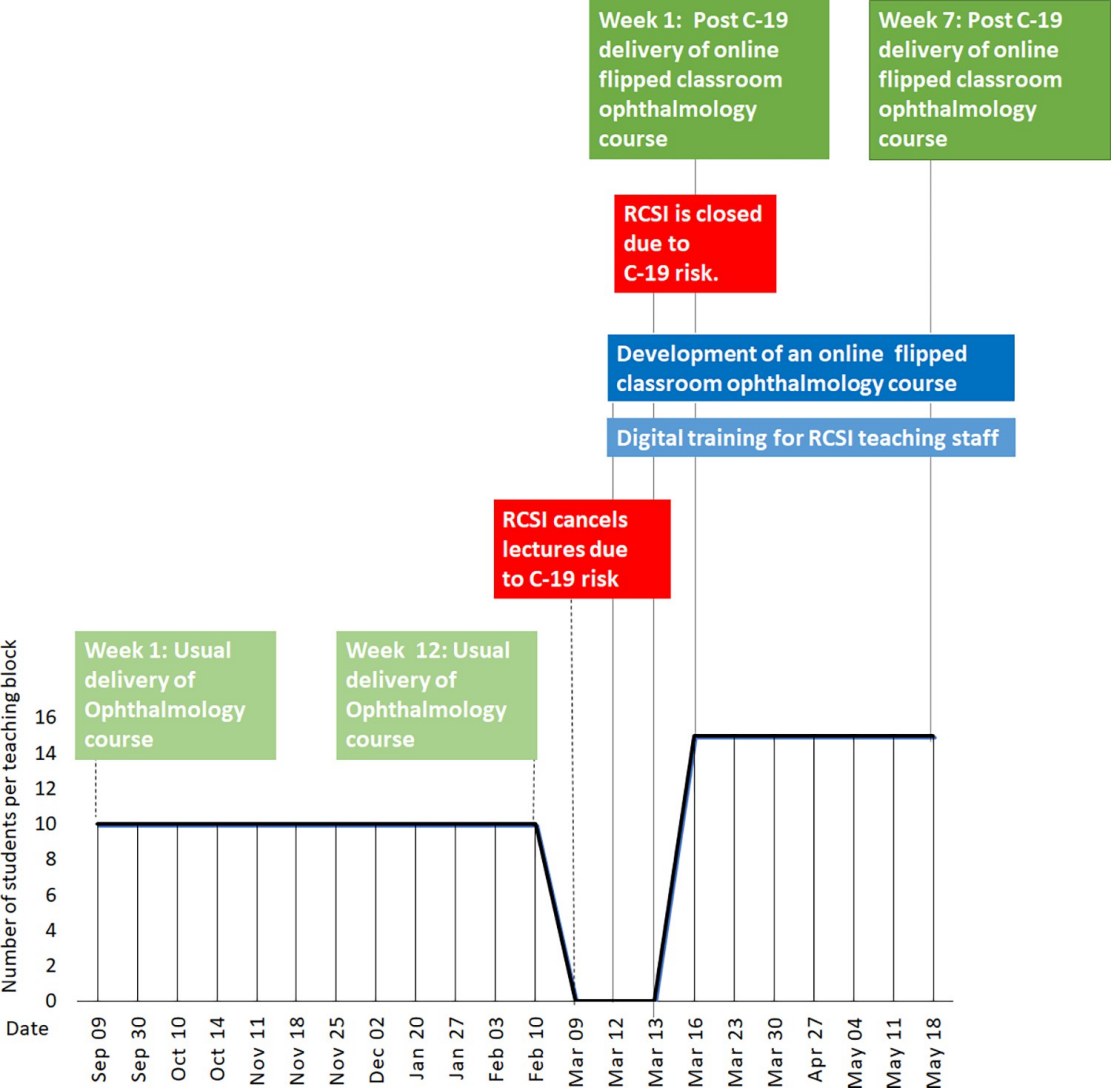
Timeline of changes to delivery of the ophthalmology course in response to COVID-19.

All students and faculty involved in the delivery of this module were invited to participate. Participants (243 students) in this study were 4^th^ year senior cycle medical students enrolled in RCSI on an Ophthalmology clinical attachment that takes place 20 times during the academic year. Traditional delivery (TD) of the 4^th^ year ophthalmology clinical attachment began on the 23^rd^ September 2019 in the Royal Victoria Eye and Ear Hospital (RVEEH) and finished 09^th^ March 2020. As a result of the global pandemic, an online distance module was devised. Students engaged in an online flipped classroom (OFC) which commenced on March 16^th^ 2020 and finished on the 22^nd^ May 2020. Faculty members (n = 5) were involved in both TD and OFC between the 23^rd^ September 2019 and the 22^nd^ May 2020 (Fig 1). As this is a natural experiment, two groups were spontaneously created; Group 1 = TD and Group 2 = OFC group.

### Traditional delivery (TD) group (pre COVID-19 usual ophthalmology module)

Students (129 students) attended didactic lectures (Red eye, Sudden loss of vision, Gradual loss of vision, Ocular trauma) prior to commencing their clinical attachment. Students were assigned into groups by the SARA (Student, Academic & Regulatory Affairs) office RCSI (10–12 in each) before commencing their clinical attachment week. The traditional ophthalmology module consisted of a review of pre-recorded videos of ophthalmic examinations performed by ophthalmologists in the Royal Victoria Eye and Ear Hospital (RVEEH) on patients and simulated patients. Students attended clinical tutorials (Cataract, Glaucoma, Diabetic Retinopathy, Age-related Macular Degeneration (AMD)), which consisted of 60-min small group teaching sessions (face-to-face lectures with 15-minute question and answer session) led by an Ophthalmologist. Students then engaged in 60-min of patient led teaching which consisted of taking patient history, reading patient charts, examining patients etc. Students also engaged in practical skills sessions (Patient Based Teaching, Clinical Skills, Outpatients Department) and peer led teaching sessions, where students collaboratively presented in groups of 2 or 3 a joint presentation on a topic assigned at the beginning of the week. Knowledge was tested upon completion of the module via a multiple- choice question (MCQ) exam. Clinical Competency (skills) were assessed by practical examination of fundoscopy skills.

### Online Flipped Classroom (OFC) group (during COVID-19 online ophthalmology module)

Students (114 students) were assigned into groups by the SARA office RCSI (12–18 in each) before attending a week long online ophthalmology course facilitated by ophthalmology department from the RVEEH. This week consisted of an online flipped classroom pedagogical approach where students reviewed pre-recorded lecture content and slide sets of the lecture material, which were made available in advance of online interactive classroom sessions. The small group didactic lectures were replaced by PowerPoint presentations with recorded audio. Clinical tutorials which were small group teaching sessions followed by patient encounters in the pre-COVID19 traditional delivery course were converted to interactive BBc tutorials on-line. Specifically students were asked to watch the pre-recorded video lectures (Cataract, Glaucoma, Diabetic Retinopathy, AMD) online in advance of the one hour interactive session led by an Ophthalmologist. This was supplemented by slide sets of the didactic lecture material without audio. After the pre-class lecture, students attended a synchronous, online, live interactive session on the same topic. These BBc sessions included problem solving, clinical vignettes and MCQs relating to the recorded lecture. Facilitators prepared clinical cases and related MCQs that addressed learning outcomes and promoted engagement for use during the interactive online session. The facilitator encouraged problem solving using the poll feature of BBc which encouraged both discussion and active learning. To facilitate a student-centred Peer Assisted Learning (PAL) pedagogical approach [27] students were provided with details on how to prepare PowerPoint presentations with audio for online peer-led teaching sessions. Knowledge was tested upon completion of the module via a multiple- choice question (MCQ) exam. Table 1 details the Red eye session format for TD versus OFC groups. A similar format was used for all topics delivered as part of the OFC approach.

**Table 1:** Format of Red eye session comparing before, during and after class activities for the traditional delivery of the ophthalmology module and the online flipped classroom delivery.

	Traditional Delivery	Online Flipped classroom
**Before class**	· Provided with timetable for the module	· Provided with an introductory email familiarising students with facilitators and course content for the week.
	· Provided with learning objectives and lecture handouts.	· Provided with guide to the online teaching delivery platform–BBc.
		· Provided learning objectives, pre-recorded lectures (with audio) and corresponding slides sets.
		· Read the relevant pre-session material provided on the course page.
		· Provided with a link to attend interactive session.
**During class**	· Listen to lecture and take notes (45 mins)	· 10 minute review and Q & A session on how to approach the history and exam in a red eye patient.
** **	· Q & A session (15 mins)	· Facilitator provides description of 5 common clinical scenarios.
	· 60 min patient led teaching–taking patient history, reading patient charts, examining patients etc	· Interactive progression through history and exam and management plan- facilitated by asking the group to provide the answers
** **		· Students are provided with written vignette then some clinical images
** **		· Students are presented with 8 MCQ questions and provide the correct answer using a poll function in BBc
** **		· Description of the other causes of red eye supported by clinical images (5–10 mins)
** **		· Summary of the class, Q & A session (15 mins)
**After class**		Anonymous feedback from students using shared whiteboard function in collaborate
**MCQ exam**		

### Instrument and data collection

The majority of studies investigating perceptions of and satisfaction with FC utilise a questionnaire (either standardised or in house) to garner feedback from the students [13]. The CEQ is utilised annually by universities in Australia and the UK, among undergraduate students, to determine student satisfaction and to identify areas for improvement [28, 29]. There is a significant body of evidence in the literature supporting the reliability and validity of this questionnaire in higher education [29–35]. Observable differences in respondent scores in diverse fields of study or between institutions offering similar programmes in the same field demonstrates the discriminant validity of the CEQ [34]. In relation to investigations specifically focussing on FC and ophthalmology students Tang, Lin and Zhu *et al* have utilised the CEQ [7, 8, 11], which Broomfield *et al* determined was an appropriate instrument for course evaluation in medical education [32]. To date Paul Ramsden’s CEQ exists in three different formats: the CEQ23; CEQ30; and CEQ36. Each item of the questionnaire is answered using a standard 5-point Likert scale where the levels of agreement ranged from “strongly agree” (scoring a “1”) to “strongly disagree” (scoring a “5”). The CEQ36 measures six constructs established as important learning environment features within the context of higher education [29–31]:

- Good teaching (GT)
- Clear goals and standards (CG)
- Appropriate assessment (AA)
- Appropriate workload (AW)
- Generic skills (GS)
- Emphasis on Independence (IN)

To investigate key stakeholders’ perceptions of and satisfaction with the online flipped classroom, all students (n = 243) and faculty (n = 5) were invited to complete the CEQ36 online via Survey Monkey.

Additionally, final anonymised MCQ exam scores were obtained for each student in the study.

### Statistical analysis

Descriptive statistics were used to describe the characteristics of the two groups and Chi-square test/Fisher exact test, or independent samples t test used to explore differences between the groups. The scores of the MCQ final exam were compared using independent samples t test. The questionnaire data given to students were analysed using Mann-Whitney-U tests, to explore potential differences between the groups. Data was collected from each staff member in relation to both teaching methods and hence the staff questionnaire data were analysed using Wilcoxon signed rank tests. No adjustment was made for multiple comparisons. During analysis responses for the ‘agree’ and ‘strongly agree’ categories were combined, similarly responses for ‘disagree’ and ‘strongly disagree’ category were combined. All statistical analyses were performed in GraphPad Prism V5 or Stata v13.

### Digital training

Blackboard Collaborate (BBc) has previously been shown to have utility as a platform to support nursing students placement learning. Several studies have highlighted the importance training to develop student’s digital literacy to facilitate student engagement with this form of technology [36, 37]. To support this guides to the use of BBc were prepared and provided to the students ahead of the online module. Digital training was provided to ophthalmology faculty along with support guides for the use of the BBc platform.

## Results

243 undergraduate medical students who received TD (n = 129) or OFC (n = 114) delivery of the ophthalmology clinical attachment were invited to participate. All students in the TD and OFC attended in person or online, completed the module and MCQ examination. Twenty-three students in the TD group and 28 students in the OFC group agreed to take part in the study and completed an online CEQ. This represents an overall participation rate of 17.8% for the TD group and 24.6% for the OFC group. All faculty (n = 5) delivered both the TD and the OFC module and completed the questionnaire giving a response rate of 100%. The demographic distribution of the participants is presented in Table 2. No gender difference was observed between TD and FC groups when compared to the class as a whole (column 1–3). Overall analysis of the demographic data indicated that a greater proportion of younger females participated in the online survey overall and specifically in the TD cohort, while the OFC survey participants were more presentative of the class as a whole.

**Table 2:** Demographic information of students who participated in traditional delivery and online flipped classroom.

	Student Groups			Survey Responders		
	All students (n)	Traditional delivery (n)	Online flipped classroom (n)	All survey responders (n) %	Traditional delivery (n) %	Online flipped classroom (n) %
Number of students	243	129	114	51 (21%)	23 (17.8%)	28 (24.6%)
Gender						
Male	114 (47.1%)	61 (47.3%)	53 (45.6%)	16 (35.3%)	3 (13%) **	13 (46.4%)
Female	129 (53.3%)	68 (52.7%)	61 (53.5%)	35 (64.7%)	20 (87%) **	15 (53.6%)
Age (years old), mean ± SD	26.6±3.6	26.7±4.1	26.5±2.8	25.2±1.9*a	24.7±1.1*b	25.6±2.3

** p = 0.0017 comparing gender distribution for all students with students in the TD group who participated in the online survey. Comparisons made using the Fisher’s exact test.

a: * p = 0.0104 comparing age of all students with those who participated in the online survey.

b: * p = 0.0189 comparing age of all students with students in the TD group who participated in the online survey. Age comparisons were conducted using Mann-Whitney-U tests.

### Student perceptions

Table 3 summarizes the responses from the students regarding the six constructs established as important learning environment features within the context of higher education: Good Teaching (GT), Generic Skills (GS), Appropriate Assessment (AA), Appropriate Workload (AW), Clear Goals and Standards (CG), Emphasis on Independence (IN) [29–31]. We observed significant differences between the responses of the TD and OFC groups regarding the learning experience, perceived value of the flipped classroom, teaching process, and the evaluation system.

**Table 3:** Comparison of students’ perspectives between traditional delivery and online flipped classroom.

STUDENTS	Strongly agree	Agree	Neither agree nor disagree	^Disagree^	Strongly disagree	p-value	Bonferroni corrected p-value	Strongly agree & agree	Disagree & Strongly disagree
**Good Teaching scale**									
Q4. The teaching staff of this course motivate students to do their best work. (FC)									
TD	43.48%	52.^1^7%	0.00%	4.35%	0.00%	0.010	0.374	95.65%	4.35%
OFC	25.00%	39.29%	25.00%	7.14%	3.57%			64.29%	10.71%
Q9. Staff here put a lot of time into commenting on students’ work.									
TD	4.35%	65.22%	17.39%	8.70%	4.35%	0.076	0.999	69.57%	13.05%
OFC	7.14%	39.29%	21.43%	25.00%	7.14%			46.43%	32.14%
Q20. The staff make a real effort to understand difficulties students may be having with their work.									
TD	8.70%	56.52%	17.39%	17.39%	0.00%	0.226	0.999	65.22%	17.39%
OFC	7.14%	42.86%	17.86%	25.00%	7.14%			50.00%	32.14%
Q22. Teaching staff here normally give helpful feedback on how you are going.									
TD	17.39%	65.22%	4.35%	8.70%	4.35%	0.041	0.999	82.61%	13.05%
OFC	0.00%	53.57%	17.86%	17.86%	10.71%			53.57%	28.57%
Q23. Our lecturers are extremely good at explaining things to us.									
TD	17.39%	60.87%	17.39%	4.35%	0.00%	0.609	0.999	78.26%	4.35%
OFC	14.29%	57.14%	25.00%	0.00%	3.57%			71.43%	3.57%
Q25. Teaching staff here work hard to make subjects interesting.									
TD	8.70%	69.57%	17.39%	4.35%	0.00%	0.345	0.999	78.27%	4.35%
OFC	7.14%	60.71%	17.86%	10.71%	3.57%			67.85%	14.28%
Q31. Staff show no real interest in what students have to say									
TD	0.00%	13.04%	17.39%	52.17%	17.39%	0.461	1.000	13.04%	69.56%
OFC	7.14%	14.29%	17.86%	50.00%	10.71%			21.43%	60.71%
Q33. This course really tries to get the best out of all its students.									
TD	0.00%	56.52%	34.78%	8.70%	0.00%	0.230	1.000	56.52%	8.70%
OFC	7.14%	35.71%	35.71%	10.71%	10.71%			42.85%	21.42%
**Clear Goals and Standards scale**									
Q1. It’s always easy here to know the standard of work expected.									
TD	17.39%	69.57%	4.35%	8.70%	0.00%	0.046	1.000	86.96%	8.70%
OFC	10.71%	50.00%	17.86%	10.71%	10.71%			60.71%	21.42%
Q8. You usually have a clear idea of where you’re going and what’s expected of you.									
TD	13.04%	56.52%	13.04%	17.39%	0.00%	0.844	1.000	69.56%	17.39%
OFC	7.14%	60.71%	10.71%	14.29%	7.14%			67.85%	21.43%
Q18. It’s often hard to discover what’s expected of you in this course.									
TD	4.35%	30.43%	4.35%	56.52%	4.35%	0.446	1.000	34.78%	60.87%
OFC	10.71%	28.57%	14.29%	46.43%	0.00%			39.28%	46.43%
Q24. The aims and objectives of this course are NOT made very clear.									
TD	8.70%	13.04%	21.74%	47.83%	8.70%	0.966	1.000	21.74%	56.53%
OFC	7.14%	14.29%	21.43%	39.29%	17.86%			21.43%	57.15%
Q35. The staff here make it clear right from the start what they expect from students.									
TD	8.70%	60.87%	21.74%	8.70%	0.00%	0.196	0.999	69.57%	8.70%
OFC	7.14%	46.43%	25.00%	14.29%	7.14%			53.57%	21.43%
**Generic Skills scale**									
Q2. This course has helped me to develop my problem-solving skills. (FC)									
TD	13.04%	60.87%	21.74%	4.35%	0.00%	0.048	0.999	73.91%	4.35%
OFC	3.57%	46.43%	25.00%	21.43%	3.57%			50.00%	25.00%
Q6. This course has sharpened my analytic skills.									
TD	0.00%	52.17%	39.13%	8.70%	0.00%	0.194	0.999	52.17%	8.70%
OFC	7.14%	35.71%	25.00%	25.00%	7.14%			42.85%	32.14%
Q11. This course has helped develop my ability to work as a team member. (FC)									
TD	0.00%	52.17%	21.74%	21.74%	4.35%	0.110	0.999	52.17%	26.09%
OFC	3.57%	21.43%	39.29%	28.57%	7.14%			25.00%	35.71%
Q12. As a result of doing this course, 1 feel more confident about tackling unfamiliar problems. (FC)									
TD	4.35%	47.83%	30.43%	13.04%	4.35%	0.408	0.999	52.18%	17.39%
OFC	3.57%	35.71%	39.29%	17.86%	3.57%			39.28%	21.43%
Q13. This course has improved my written communication skills. (FC)									
TD	0.00%	34.78%	26.09%	30.43%	8.70%	0.467	0.999	34.78%	39.13%
OFC	7.14%	25.00%	14.29%	42.86%	10.71%			32.14%	53.57%
Q28. This course has helped me develop the ability to plan my own work. (FC)									
TD	4.35%	34.78%	39.13%	21.74%	0.00%	0.387	0.999	39.13%	21.74%
OFC	7.14%	28.57%	25.00%	35.71%	3.57%			35.71%	39.28%
**Appropriate Assessment scale**									
Q7. Lecturers here frequently give the impression they have nothing to learn from students.									
TD	0.00%	13.04%	30.43%	56.52%	0.00%	0.102	0.999	13.04%	56.52%
OFC	7.14%	28.57%	25.00%	32.14%	7.14%			35.71%	39.28%
Q10. To do well on this course all you really need is a good memory.									
TD	4.35%	34.78%	4.35%	52.17%	4.35%	0.326	0.990	39.13%	56.52%
OFC	3.57%	35.71%	28.57%	32.14%	0.00%			39.28%	32.14%
Q17. Staff seem more interested in testing what you’ve memorised than what you’ve understood.									
TD	0.00%	30.43%	8.70%	56.52%	4.35%	0.575	0.999	30.43%	60.87%
OFC	3.57%	28.57%	17.86%	35.71%	14.29%			32.14%	50.00%
Q26. Too many staff ask us questions just about facts.									
TD	0.00%	26.09%	21.74%	47.83%	4.35%	0.690	0.999	26.09%	52.18%
OFC	7.14%	14.29%	21.43%	50.00%	7.14%			21.43%	57.14%
Q29. Feedback on student work is usually provided ONLY in the form of marks and grades.									
TD	17.39%	26.09%	8.70%	47.83%	0.00%	0.490	0.999	43.48%	47.83%
OFC	17.86%	28.57%	21.43%	32.14%	0.00%			46.43%	32.14%
Q32. It would be possible to get through this course just by working hard around exam times.									
TD	4.35%	39.13%	17.39%	34.78%	4.35%	0.160	0.999	43.48%	39.13%
OFC	17.86%	46.43%	10.71%	17.86%	7.14%			64.29%	25.00%
**Appropriate Workload scale**									
Q5. The workload is too heavy. (FC)									
TD	8.70%	17.39%	21.74%	47.83%	4.35%	0.002	0.070	26.09%	52.18%
OFC	0.00%	0.00%	10.71%	85.71%	3.57%			0.00%	89.28%
Q14. It seems to me that the syllabus tries to cover too many topics.									
TD	8.70%	13.04%	17.39%	52.17%	8.70%	0.298	0.999	21.74%	60.87%
OFC	3.57%	10.71%	10.71%	67.86%	7.14%			14.28%	75.00%
Q19. We are generally given enough time to understand the things we have to learn.									
TD	4.35%	43.48%	17.39%	34.78%	0.00%	0.555	0.999	47.83%	34.78%
OFC	0.00%	53.57%	21.43%	25.00%	0.00%			53.57%	25.00%
Q27. There’s a lot of pressure on you as a student here.									
TD	4.35%	17.39%	30.43%	39.13%	8.70%	0.081	0.999	21.74%	47.83%
OFC	10.71%	28.57%	35.71%	25.00%	0.00%			39.28%	25.00%
Q36. The sheer volume of work to be got through in this course means you can’t comprehend it all thoroughly.									
TD	4.35%	21.74%	17.39%	52.17%	4.35%	0.645	0.999	26.09%	56.52%
OFC	3.57%	14.29%	21.43%	53.57%	7.14%			17.86%	60.71%
**Emphasis on Independence scale**									
Q3. There are few opportunities to choose the particular areas you want to study.									
TD	13.04%	43.48%	34.78%	8.70%	0.00%	0.133	0.999	56.52%	8.70%
OFC	14.29%	28.57%	25.00%	32.14%	0.00%			42.86%	32.14%
Q15. The course has encouraged me to develop my own academic interests as far as possible.									
TD	0.00%	56.52%	34.78%	8.70%	0.00%	0.439	0.999	56.52%	8.70%
OFC	0.00%	50.00%	28.57%	17.86%	3.57%			50.00%	21.43%
Q16. Students have a great deal of choice over how they are going to learn in this course.									
TD	0.00%	47.83%	30.43%	21.74%	0.00%	0.018	0.659	47.83%	21.74%
OFC	7.14%	17.86%	17.86%	53.57%	3.57%			25.00%	57.14%
Q21. Students here are given a lot of choice in the work they have to do.									
TD	0.00%	21.74%	43.48%	34.78%	0.00%	0.011	0.407	21.74%	34.78%
OFC	3.57%	17.86%	17.86%	53.57%	7.14%			21.43%	60.71%
Q30. We often discuss with our lecturers or tutors how we are going to learn in this course.									
TD	0.00%	34.78%	26.09%	34.78%	4.35%	0.064	0.999	34.78%	39.13%
OFC	0.00%	25.00%	3.57%	53.57%	17.86%			25.00%	71.43%
Q34. There’s very little choice in this course in the ways you are assessed.									
TD	8.70%	30.43%	43.48%	17.39%	0.00%	<0.001	0.004	39.13%	17.39%
OFC	32.14%	57.14%	10.71%	0.00%	0.00%			89.28%	0.00%
Q37. Overall, I am satisfied with the quality of this course.									
TD	21.74%	56.52%	8.70%	13.04%	0.00%	0.784	0.999	78.26%	13.04%
OFC	14.29%	67.86%	3.57%	7.14%	7.14%			82.15%	14.28%

Notes

1. Strongly Agree & Agree categories joined for analysis

2. Strongly Disagree & Disagree categories joined for analysis

### Good teaching scale

There was no evidence of a difference in student perceptions about the amount of time staff spent commenting on their work (Q9) or the effort staff made to understand student difficulties (Q20). Both the TD and OFC group felt that lecturers were good at explaining (Q23) and worked hard to make their topics interesting (Q25). Furthermore, the TD and OFC students felt that faculty had an interest in what students had to say (Q31) and that they tried to get the best out of all the students (Q33). However, compared to the TD group who attended the on-site traditional clinical attachment, students felt that the OFC approach did not motivate students to do their best (Q4, P = 0.01) or provide adequate feedback on how they were doing (Q22, P = 0.041) on the GT scale.

### Clear goals and standards scale

There was no evidence of a difference in student perceptions on the goals and standards (CG) scale specifically about what was expected from them (Q8 & 18), about the objectives of the course (Q24) and faculty expectations of students being made clear (Q35). The OFC group were significantly less satisfied with the CG. Specifically, students felt it was harder to determine what standard of work was expected (Q1, P = 0.046).

#### Generic skills scale

There was no evidence of a difference in student perceptions about the capacity for the TD or OFC course to sharpen their analytic skills (Q6), develop their ability to work as a team member (Q11), improve confidence about tackling unfamiliar problems (Q12), improve communication skills (Q13) or develop their ability to plan work (Q28). On the GS scale students felt that the OFC approach was significantly less beneficial in terms of helping develop their problem skills when compared to the TD group (Q2, P = 0.048).

#### Appropriate assessment scale

There was no evidence of a difference between the TD and OFC group in student perceptions of the impression faculty gave about learning from students (Q7), that a good memory is all that is required to do well on the course (Q10), and that staff are more interested in testing what students have memorised (Q17). There was also no evidence of a difference between student perceptions from the two groups in relation to asking too many questions about facts (Q26). However, there was difference between the TD and OFC group in their perceptions of the form that feedback was given (Q29) or that just by working hard around exam times they could get through the course (Q32).

#### Appropriate workload scale

There was no evidence of a difference in student perceptions in relation to the amount of topics covered in the syllabus (Q14), the amount of time given to learn (Q19), the pressure felt by students (Q27) or how the volume of work affects comprehension of topics (Q36). Interestingly, on the AW scale there was a significant difference in feeling about the workload being too heavy in TD group compared to the OFC group (Q5, P = 0.002).

#### Emphasis on independence scale

There was no evidence of a difference in student perceptions between the TD and OFC group on the IN scale regarding opportunities to choose the particular areas you want to study (Q3), that the course encouraged them to pursue their academic interests (Q15) or the opportunities to discuss faculty how they were going to learn in this course (Q30). Analysis of the IN scale indicates that students were dissatisfied with the level of choice afforded by the OFC. Students within the TD group found that they had little choice over how they would learn (Q16, P = 0.018) or over the work they had to do (Q21, P = 0.011). Additionally, students in the OFC group felt that they had little choice in the way they were assessed compared to the TD group (Q34, P > 0.001).

#### Questions regarding the value of the flipped classroom

Previous studies have highlighted questions within the CEQ survey, which provide insights into the perceived value of the flipped classroom [7], indicated by FC in Table 3. The FC scale questions overlap with the GT and GS scale; specifically questions 2, 4, 5, 11, 12, 13 and 28. Student survey responses indicated a lack of satisfaction with the online flipped classroom approach. As mentioned above students in the TD group felt that staff did less to motivate them (Q2, P = 0.048) and that there were less opportunities to improve their problem-solving skills (Q4, P = 0.01). While not reaching statistical significance within the FC scale a reduced percentage of students within the OFC group agreed that the online flipped classroom helped develop their ability to work as a team member compared to the TD group (Q11, 52.17% v 25% agree TD v OFC), tackle unfamiliar problems (Q12, 52.18% v 39. 28% agree TD v OFC) or develop their ability to plan their own work (Q28, 21.74% v 39.28% disagree TD v OFC). Overall, study participants showed no preference for traditional delivery of the ophthalmology over the online flipped classroom approach (Q 37). When asked to rate the statement “Overall, I am satisfied with the quality of this course” there was no evidence of a difference in the rating between the TD and the OFC group.

#### Comparison of faculty perspectives between the traditional delivery and the online flipped classroom

Table 4 summarises the feedback from the faculty who taught the didactic and clinical–skills ophthalmology module on site and delivered the OFC ophthalmology module. There was no evidence of differences across the five scales. We compared trends in agreement from Face-to-Face teaching to online flipped classroom teaching for faculty, we defined changes as; agreement increased, stayed the same or decreased from face-to-face to the online approach (Table 5). On the GS scale, this analysis revealed that more faculty agreed that they put a lot of time into commenting on students work (Q9) and providing feedback (Q22) for the online versus the face-to-face approach. CS scale showed faculty agreed that it was easy to determine the standard of work expected (Q1) while the aims and objectives of this course were not made very clear (Q 24) for the OFC versus the TD approach. Comparison of the GS scale indicated that faculty perceived that the OFC approach supported improvements in student problem solving skills (Q2), analytic skills (Q6) and ability to tackle unfamiliar problems (Q12). An increased number of faculty disagreed that lecturers asked questions specifically about facts (Q26) on the AA scale for OFC approach versus traditional face-to-face teaching. Comparison of trends for the IN scale indicated that faculty felt the online flipped classroom approach had increased opportunities for students to choose particular areas of study (Q3), that the course encouraged students to develop their own academic interests (Q15) and provided more options regarding how students were going to learn on the course (Q16).

**Table 4:** Comparison of faculty perspectives between traditional classroom and online flipped classroom ophthalmology.

Faculty	Strongly agree	Agree	Neither agree nor disagree	Disagree	Strongly disagree	p-value	Bonferroni corrected p-value
**Good Teaching scale**							
Q4. The teaching staff of this course motivate students to do their best work.							
TD	60.00%	40.00%	0.00%	0.00%	0.00%	na	na
OFC	40.00%	60.00%	0.00%	0.00%	0.00%		
Q9. Staff here put a lot of time into commenting on students’ work.							
TD	0	80	0	20	0	na	na
OFC	40	40	0	20	0		
Q20. The staff make a real effort to understand difficulties students may be having with their work.							
TD	20	80	0	0	0	0.3173	0.999
OFC	40	40	20	0	0		
Q22. Teaching staff here normally give helpful feedback on how you are going.							
TD	0	80	20	0	0	0.1573	0.999
OFC	40	20	20	20	0		
Q23. Our lecturers are extremely good at explaining things to us.							
TD	60	40	0	0	0	0.3173	0.999
OFC	40	40	20	0	0		
Q25. Teaching staff here work hard to make subjects interesting.							
TD	60	40	0	0	0	na	na
OFC	40	60	0	0	0		
Q31. Staff show no real interest in what students have to say							
TD	0	0	0	60	40	na	na
OFC	0	0	0	40	60		
Q33. This course really tries to get the best out of all its students.							
TD	20	80	0	0	0	na	na
OFC	20	80	0	0	0		
**Clear Goals and Standards scale**							
Q1. It’s always easy here to know the standard of work expected.							
TD	20	60	20	0	0	0.999	0.999
OFC	20	60	20	0	0		
Q8. You usually have a clear idea of where you’re going and what’s expected of you.							
TD	20	80	0	0	0	na	na
OFC	40	60	0	0	0		
Q18. It’s often hard to discover what’s expected of you in this course.							
TD	0	0	0	80	20	na	na
OFC	0	0	0	80	20		
Q24. The aims and objectives of this course are NOT made very clear.							
TD	0	0	0	40	60	na	na
OFC	0	0	0	60	40		
Q35. The staff here make it clear right from the start what they expect from students.							
TD	20	80	0	0	0	na	na
OFC	20	80	0	0	0		
**Generic Skills scale**							
Q2. This course has helped me to develop my problem-solving skills.							
TD	0	60	40	0	0	0.1573	0.999
OFC	40	60	0	0	0		
Q6. This course has sharpened my analytic skills.							
TD	0	80	0	20	0	0.3173	0.999
OFC	40	60	0	0	0		
Q11. This course has helped develop my ability to work as a team member.							
TD	20	60	20	0	0	0.0833	0.999
OFC	20	20	40	20	0		
Q12. As a result of doing this course, 1 feel more confident about tackling unfamiliar problems.							
TD	0	80	20	0	0	0.999	0.999
OFC	20	60	20	0	0		
Q13. This course has improved my written communication skills.							
TD	40	60	0	0	0	0.1599	0.999
OFC	40	20	20	20	0		
Q28. This course has helped me develop the ability to plan my own work.							
TD	20	60	20	0	0	0.999	0.999
OFC	20	60	20	0	0		
**Appropriate Assessment scale**							
Q7. Lecturers here frequently give the impression they have nothing to learn from students.							
TD	0	0	20	60	20	0.3173	0.999
OFC	0	0	0	60	40		
Q10. To do well on this course all you really need is a good memory.							
TD	20	0	0	60	20	0.8759	0.999
OFC	0	0	20	60	20		
Q17. Staff seem more interested in testing what you’ve memorised than what you’ve understood.							
TD	0	0	0	60	40	na	na
OFC	0	0	0	60	40		
Q26. Too many staff ask us questions just about facts.							
TD	20	0	40	20	20	0.0881	0.999
OFC	0	0	0	80	20		
Q29. Feedback on student work is usually provided ONLY in the form of marks and grades.							
TD	0	0	0	80	20	0.3173	0.999
OFC	0	0	20	60	20		
Q32. It would be possible to get through this course just by working hard around exam times.							
TD	0	40	20	20	20	0.3938	0.999
OFC	0	20	0	60	20		
**Appropriate Workload scale**							
Q5. The workload is too heavy.							
TD	0	0	0	80	20	na	na
OFC	0	0	0	80	20		
Q14. It seems to me that the syllabus tries to cover too many topics.							
TD	0	40	0	40	20	na	na
OFC	0	40	0	60	0		
Q19. We are generally given enough time to understand the things we have to learn.							
TD	20	60	20	0	0	0.3173	0.999
OFC	20	60	0	20	0		
Q27. There’s a lot of pressure on you as a student here.							
TD	0	0	20	60	20	na	na
OFC	0	0	20	60	20		
Q36. The sheer volume of work to be got through in this course means you can’t comprehend it all thoroughly.							
TD	0	20	0	60	20	na	na
OFC	0	20	0	60	20		
**Emphasis on Independence scale**							
Q3. There are few opportunities to choose the particular areas you want to study.							
TD	20	40	20	20	0	0.4922	0.999
OFC	20	20	20	40	0		
Q15. The course has encouraged me to develop my own academic interests as far as possible.							
TD	0	40	40	20	0	0.5673	0.999
OFC	20	20	20	40	0		
Q16. Students have a great deal of choice over how they are going to learn in this course.							
TD	20	0	20	60	0	0.1599	0.999
OFC	40	20	0	40	0		
Q21. Students here are given a lot of choice in the work they have to do.							
TD	20	40	20	20	0	0.8759	0.999
OFC	20	40	0	20	20		
Q30. We often discuss with our lecturers or tutors how we are going to learn in this course.							
TD	20	40	20	0	20	0.3173	0.999
OFC	20	60	20	0	0		
Q34. There’s very little choice in this course in the ways you are assessed.							
TD	20	20	20	40	0	0.3173	0.999
OFC	20	40	0	20	20		
Q37. Overall, I am satisfied with the quality of this course.							
TD	0	100	0	0	0	na	na
OFC	40	60	0	0	0		

**Table 5:** Comparison of faculty trends in agreement from face-to-face teaching to online flipped classroom.

Question	Scale	Agreement increased from Face-to-Face teaching to online flipped classroom	Agreement stayed the same (Face to Face teaching and online flipped classroom)	Agreement decreased from Face-to-Face teaching to online flipped classroom
	**Good Teaching scale**			
4	The teaching staff of this course motivate students to do their best work.	0 (0%)	4 (80%)	1 (20%)
9	Staff here put a lot of time into commenting on students’ work.	2 (40%)	3 (60%)	0 (0%)
20	The staff make a real effort to understand difficulties students may be having with their work.	1 (20%)	3 (60%)	1 (20%)
22	Teaching staff here normally give helpful feedback on how students are going.	2 (40%)	1 (20%)	2 (40%)
23	The lecturers are extremely good at explaining things to students.	0 (0%)	3 (60%)	2 (40%)
25	Teaching staff here work hard to make subjects interesting.	0 (0%)	4 (80%)	1 (20%)
31	Staff show no real interest in what students have to say.	0 (0%)	4 (80%)	1 (20%)
33	This course really tries to get the best out of all its students.	0 (0%)	5 (100%)	0 (0%)
	**Clear Goals and Standards scale**			
1	It’s always easy here to know the standard of work expected.	2 (40%)	1 (20%)	2 (40%)
8	Students usually have a clear idea of where they are going and what’s expected of them.	1 (20%)	4 (80%)	0 (0%)
18	It’s often hard to discover what’s expected of students in this course.	0 (0%)	5 (100%)	0 (0%)
24	The aims and objectives of this course are NOT made very clear.	2 (40%)	2 (40%)	1 (20%)
35	The staff here make it clear right from the start what they expect from students.	1 (20%)	3 (60%)	1 (20%)
	**Generic Skills scale**			
2	This course helps to develop students problem-solving skills.	3 (60%)	2 (40%)	0 (0%)
6	This course sharpens students analytic skills.	2 (40%)	3 (60%)	0 (0%)
11	This course helps develop students ability to work as a team member.	0 (0%)	2 (40%)	3 (60%)
12	As a result of doing this course, students feel more confident about tackling unfamiliar problems.	2 (40%)	2 (40%)	1 (20%)
13	This course improves students communication skills.	1 (20%)	2 (40%)	2 (40%)
28	This course helps students develop the ability to plan their own work.	1 (20%)	3 (60%)	1 (20%)
	**Appropriate Assessment scale**			
7	Lecturers here frequently give the impression they have nothing to learn from students.	0 (0%)	4 (80%)	1 (20%)
10	To do well on this course all you really need is a good memory.	1 (20%)	3 (60%)	1 (20%)
17	Staff seem more interested in testing what students have memorised than what they’ve understood.	1 (20%)	3 (60%)	1 (20%)
26	Too many staff ask questions just about facts.	0 (0%)	2 (40%)	3 (60%)
29	Feedback on student work is usually provided ONLY in the form of marks and grades.	1 (20%)	4 (80%)	0 (0%)
32	It would be possible to get through this course just by working hard around exam times.	1 (20%)	2 (40%)	2 (40%)
	**Appropriate Workload scale**			
5	The workload is too heavy.	0 (0%)	5 (100%)	0 (0%)
14	It seems to me that the syllabus tries to cover too many topics.	0 (0%)	4 (80%)	1 (20%)
19	Students are generally given enough time to understand the things they have to learn.	0 (0%)	4 (80%)	1 (20%)
27	There’s a lot of pressure on you as a student here.	1 (20%)	3 (60%)	1 (20%)
36	The sheer volume of work to be got through in this course means students can’t comprehend it all thoroughly.	0 (0%)	5 (100%)	0 (0%)
	**Emphasis on Independence scale**			
3	There are few opportunities to choose the particular areas students want to study.	1 (20%)	1 (20%)	3 (60%)
15	The course encourages students to develop their own academic interests as far as possible.	2 (40%)	1 (20%)	2 (40%)
16	Students have a great deal of choice over how they are going to learn in this course.	2 (40%)	3 (60%)	0 (0%)
21	Students here are given a lot of choice in the work they have to do.	1 (20%)	1 (20%)	3 (60%)
30	We often discuss with students how they are going to learn in this course.	1 (20%)	4 (80%)	0 (0%)
34	There’s very little choice in this course in the ways students are assessed.	1 (20%)	3 (60%)	1 (20%)
37	Overall, I am satisfied with the quality of this course.	2 (40%)	3 (60%)	0 (0%)

### Comparison of overall student performance on final multiple-choice exam

Next we compared students’ exam scores before and after the educational intervention for all students in the TD (n = 129) and OFC groups (n = 114) and students in the TD (n = 23) and OFC groups (n = 28) who responded to the survey. Students answered 20 ophthalmology multiple-choice questions (MCQ) as part of completing the course. Each question had the same weight, and the total score was converted into a 0–100 scale. Independent samples t test was used to compare the differences between the two groups. This analysis of the final exam MCQ score showed that there were no statistical differences between the TD and OFC group (P = 0.0651). Comparison of the final exam MCQ score for survey responders between the TD and OFC found no evidence of a statistical difference in the score achieved. Overall, this indicates that the OFC did not negatively influence knowledge gain.

## Discussion

The primary aim of this study was to measure stakeholder satisfaction with our usual delivery format, which previously relied on a blend of didactic lectures and clinical skills sessions compared to a revised format, which relied on a FC format to facilitate continued delivery of the ophthalmology module. It was hypothesised that as an educational intervention in response to the COVID-19 pandemic, a FC approach would not only facilitate delivery of content but result in improved or equivalent levels of student satisfaction and knowledge gain as determined by a CEQ and MCQ examination.

In pivoting from didactic face-to-face teaching to a solely online delivery of the ophthalmology module, we found that students were satisfied with the quality of this course in both the TD and the OFC groups. However, students in the OFC group were less satisfied with staff motivation of students and provision of feedback, compared to the TD group. OFC students also felt it was harder to determine what standard of work was expected and found the course less beneficial in terms of helping develop their problem-solving skills. Furthermore, students were dissatisfied with the level of choice afforded by the online FC, compared to TD group. Of note, while student responses indicated a lack of satisfaction with the OFC approach compared to traditional face-to-face teaching exam scores were consistent between both groups.

Recent reviews have examined developments in medical education interventions in the wake of the pandemic [15, 16]. These reviews found that a significant proportion of interventions focused on pivoting traditional teaching online (53%) with a significant proportion of these studies using the same teaching approaches for face-to-face teaching and their online approach. These reviews noted there was a lack of interventional outcome data in the studies reviewed. More recently, Ferrara *et al* conducted an online survey to assess the changes ophthalmology residents and fellows have experienced in ophthalmology training related to the current COVID-19 pandemic and collected responses from 32 different countries. Based on the survey responses of ophthalmology trainees’ perspectives the authors suggest that incorporation of technological educational tools, can increase the capacity to cope successfully with the current situation and continued use may improve the effectiveness of training programs in the long term [38]. However, the perspective of Irish ophthalmology students, trainees or fellows was not included in this study.

Herein the current study describes an educational intervention and uniquely provides data on the perspectives of those involved: both student and faculty satisfaction and perceptions of an online flipped classroom approach for ophthalmology teaching. We devised a remote synchronous and asynchronous module that was instigated in response to the global COVID-19 pandemic. By adopting an online flipped classroom approach and incorporating elements of Blooms taxonomy we aimed to move away from recall and enhance critical thinking and application of knowledge [3]. Overall, our results are in direct contrast to existing literature regarding the utility of a flipped classroom approach for delivering ophthalmology content to medical students. Previously it has been shown that students preferred the flipped classroom approach to the traditional lecture method as it helped them to develop problem solving, creative thinking and team working skills [7, 10]. However, we found significant dissatisfaction with the online flipped classroom approach among the OFC group. Significant levels of dissatisfaction were observed in problem solving, communication, staff motivation and provision of feedback. Previous groups examining a flipped classroom approach for ophthalmology students have reported higher levels of knowledge gain (increased exam scores) compared to the traditional teaching group [8–10]. The current study found that exam outcome was consistent with both approaches: the remote flipped classroom approach did not improve knowledge gain for the OFC group compared to the prior didactic and clinical skills-based module.

A recent review detailed the types of uncertainty faced by students, including those relating to the educational process and the global coronavirus pandemic, and explored the potential negative impact this can have on learning [39]. Unger *et al* reported that students experienced a significant level of anxiety in relation to online learning and that this anxiety persisted beyond an initial 3-week practice period [40].

Shahrvini *et al* similarly reported that students experienced anxiety with the transition of their curriculum online and perceived this mode of delivery had negatively impacted the quality of instruction [41]. Students reported satisfaction with the flexibility of remote learning, however Shahrvini *et al* suggests that removal of face-to-face teaching and adoption of a solely online program, which leads to digital fatigue, coupled with loss of practical elements of the curriculum contributed to the students negative perceptions towards online learning [41]. They suggest that video casted lectures uploaded in advance, electronic health record and telehealth training for students, in addition to training for teaching faculty to increase technological fluency should be considered to optimise remote learning curricula [41]. A survey conducted by Mishra *et al* highlights that the COVID-19 lockdown has brought with it, uncertainty, anxiety and higher stress levels among ophthalmology trainees across India due to the disruption of training program schedules [42]. Therefore, anxiety, uncertainty and digital fatigue may be factors contributing to student dissatisfaction with the online flipped classroom approach in our study.

Faculty preparedness is another aspect that may have influenced student satisfaction with our educational intervention. Overall, despite literature describing a range of innovative ways to deliver teaching in response to the pandemic, there is relatively little existing in current literature focusing on faculty development or support. Of note the BEME Guide No 63 [16] highlights three studies that describe measures used to support medical educators move to online including adapting existing programs for online delivery, curating resources, providing a webinar where best practices were shared and establishing a twitter community of practice [43–45]. Furthermore, BEME Guide No 64 [15]: described the faculty development intervention undertaken by Buckley who established a virtual faculty development session to bring together regionally dispersed teaching faculty groups together to learn with and from each other using social networking theory [46]. This virtual faculty development initiative had a range of benefits including supporting faculty to embrace the new challenges of teaching online and importantly providing skills to allow faculty to attend to student well-being, however some issues with its implementation were highlighted. Specifically, that the online webinar did not permit informal side bar conversations, that organisers should consider inclusion of smaller break out rooms to promote discussion along with additional forms of follow up commination eg email [46].

Faculty in RCSI, including those involved in ophthalmology teaching, attended an in-person training course before the closure of the campus, which aimed to increase faculty digital competence with the online delivery platform BBc as we pivoted delivery of our module online. Results from our study indicate that students felt on the ‘Good Teaching Scale’ that the faculty approach to delivery of the OFC did not motivate students to do their best or provide adequate feedback. This resulted in students feeling that the module was overall less beneficial in terms of helping them to develop their problem skills when compared to the TD group. Thus, in line with the main findings of the recent BEME reviews our results confirm that significant additional levels of support are required to support faculty to develop and implement online learning.

Additional factors potentially contributing to the overall dissatisfaction with the course include the lack of clinical experiences in the redesigned ophthalmology curriculum, reduced digital efficacy and technical issues. Kostaki *et al* found that student engagement negatively correlated with technical difficulties and home distractions [47]. Furthermore, they reported that computer self-efficacy positively contributed towards engagement as students could remedy technical difficulties [47]. Brockman *et al* compared student perceptions of an online or in-person microbiology laboratory and found that while students have positive perceptions of digital online activities there was still a significant preference for a blend of online and in-person practical activities [48].

There has been significant interest in the FC pedagogy a means to continue to deliver education during these extraordinary times [49–51]. While many researchers have indicated that this approach should be maintained long term in an effort to modernise medical education [42, 52], Fisher *et al* advised that the strategy for the flipped classroom is important, as the environment needs to facilitate learning rather than influence learning to be satisfactory for students [53].

### Change to curriculum

Our investigations and comparison with contemporary literature indicate that students prefer face-to-face teaching with practical elements. While inclusion of online technology within the curriculum is essential to enhance student digital proficiency and future proof against future disruptions, elements of the traditional curriculum are indispensable.

Therefore, future developments should include a blend of traditional classroom-based and remote learning approaches combined with in person practical elements including direct patient contact with mitigated risk. Additionally training of both faculty and students will help to increase digital proficiency and engagement as online elements are predicted to be a central feature on medical education developments.

### Study limitations

The results from our investigations should be viewed in light of the limitations of this study. One such limitation is the lack of student participation in the study resulting in poor respondent rates. The ongoing global pandemic at the time of participant recruitment is a potential factor leading to a lack of study respondents. Additionally we have identified the delay from module completion to distribution of the post-clinical attachment survey as another factor influencing study participation. However, by using the widely validated CEQ we believe that this study offers a unique and important reflection of the impact that the pandemic has had on student learning during the pandemic. Another limitation is that that the OFC did not include clinical skills training which a staple in the TD approach is. It was not possible for our department to teach clinical skills online and consequently this aspect of the clinical attachment was not assessed for the OFC groups. Future studies seeking to determine changes in student and learning and student satisfaction with the OFC for delivery of Ophthalmology content should address these limitations by inclusion of our suggested changes to the curriculum.

## Supporting information

S1 Appendix Ophthalmology module learning outcomes. (DOCX)
