# Supplementary material for: How has medical student learning changed with the pivot to online delivery of ophthalmology in the pandemic?
Source: PLoS One. 2023 Mar 30;18(3):e0282829. doi: 10.1371/journal.pone.0282829 (PMC10062639; doi:10.1371/journal.pone.0282829)
Supplement: S1 Appendix — (DOCX) [file pone.0282829.s001.docx]

**Lecture learning outcomes**

**Anatomy of the eye and visual pathway**

Senior cycle 1 2019-2020

At this end of this tutorial, you will be able to:

- Describe the anatomy and physiology of the orbit, its contents, and adnexa
- Understand and describe the components of the visual pathway
- Understand and describe the optical elements of the eye
- Explain the principles of refractive error and its related terminology

**Sudden visual disturbance**

Senior cycle 1 2019-2020

At the end of this tutorial you will be able to:

- Describe the major causes of sudden loss of vision
- Use a symptoms-based approach to taking a comprehensive vision history
- Perform the relevant examinations
- Assimilate the findings from the history and examination leading to an accurate differential diagnosis
- Make an appropriate referral to a specialist for further assessment when required

**Gradual loss of vision**

Senior cycle 1 2019-2020

At the end of this tutorial, you will be able to:

- Describe the major causes of gradual loss of vision
- Explain how changes in the cornea, lens, and retina can affect vision
- Take a comprehensive vision history
- Perform relevant examinations and investigations
- Define appropriate treatment regimens

**The red eye**

Senior cycle 1 2019-20

At the end of this tutorial, for all cases of red eye you will be able to:

- Use a symptoms-based approach to taking a comprehensive history
- Perform the relevant examination
- Assimilate the findings from the history and examination leading to an appropriate differential diagnosis
- Make a decision about the urgency of referral to a specialist and know when it is safe for a non-specialist to manage a patient with red eye
- Define appropriate treatment regimens for common causes of red eye

**Ocular trauma**

Senior cycle 1 2019-2020

- Demonstrate adequate primary care assessment and management of an ocular injury
- Discuss the identification and assessment of an intraocular foreign body
- Describe how to manage a hyphaema
- Describe the immediate management of chemical injuries
- Outline the assessment and management of a blow out fracture of the orbit

**Key Topic Tutorials**

**Diabetic retinopathy**

Senior cycle 1 2019-2020

At the end of this tutorial, you will be able to:

- Describe the basic epidemiology of diabetic retinopathy (DR) as a major cause of blindness
- Explain the basic pathology that damages the retina in DR
- Assess a patient presenting with DR, including taking a relevant history and performing the appropriate examination and investigations
- Classify DR based on the clinical findings
- Describe the management of DR including both systemic and ocular treatment
- Understand the importance of screening in diabetes

**Glaucoma**

Senior cycle 1 2019-2020

At the end of this tutorial, you will be able to:

- Describe the basic epidemiology of glaucoma as a major cause of blindness
- Explain the basic pathophysiology of glaucoma
- Classify glaucoma based on the clinical findings
- Assess a patient presenting with glaucoma, including taking a relevant history and performing the appropriate examination and investigations
- Describe the management of glaucoma

**Age related macular degeneration**

Senior cycle 1 2019-2020

At the end of this tutorial, you will be able to:

- Describe the basic epidemiology of ARMD as a major cause of blindness
- Explain the basic pathology that damages the retina in ARMD
- Assess a patient presenting with ARMD, including taking a relevant history and performing the appropriate examination and investigations
- Describe treatment regimens for ARMD and advise patients on recognised preventative measures

**Cataract**

Senior cycle 1 2019-2020

At the end of this tutorial, you will be able to:

- Describe the basic epidemiology of cataract as a major cause of vision loss
- Describe the causes of cataract
- Assess a patient presenting with cataract, including taking a relevant history and performing the appropriate examination
- Describe the indications for cataract surgery
- Describe the major causes of cataract in a child

**RCSI Senior cycle 1 Peer led teaching**

By the end of this session, learners will be able to:

1. present on a topic that supports the learning of their peers
2. demonstrate collaborative skills
3. engage in peer led feedback
